# Prevalence of gastrointestinal parasites in communal goats from different agro-ecological zones of South Africa

**DOI:** 10.14202/vetworld.2020.26-32

**Published:** 2020-01-04

**Authors:** Takalani J. Mpofu, Khathutshelo A. Nephawe, Bohani Mtileni

**Affiliations:** Department of Animal Science, Tshwane University of Technology, Private Bag X680, Pretoria 0001, South Africa

**Keywords:** *Eimeria*, *Moniezia*, strongyle, *Strongyloides papillosus*, *Trichuris* spp

## Abstract

**Aim:**

A longitudinal study was conducted to assess the epidemiology of common gastrointestinal parasite (GIP) infections affecting goats in South Africa as influenced by agro-ecological zone (AEZ), sampling season, and the age and sex of animals.

**Materials and Methods:**

A total of 288 goats (101 male and 187 female) were randomly sampled during winter and summer in areas representing four AEZs (arid: 80; semi-arid: 76; humid: 62; and dry sub-humid: 70) of South Africa. Fecal samples from each animal were collected from the rectum, and the presence of GIP eggs was determined using a modified McMaster technique. A sample was considered positive when a minimum of one GIP egg was detected under the microscope. Fecal cultures were prepared, and infective larvae were collected and identified. The data were analyzed by MiniTab17 (2017) using the FREQ procedure, and the association between the independent factors and the prevalence of various GIPs were evaluated using the Pearson Chi-square test (p<0.05).

**Results:**

The overall prevalence of GIP in the present study was 37.1%, with a mean prevalence of 30.0, 26.4, 31.1, 36.6, and 59.6% for *Eimeria* spp., *Trichuris*, *Strongyloides papillosus*, *Moniezia* spp., and strongyles, respectively. There was a significant (p<0.05) association between the prevalence of strongyles, *Trichuris*, *Moniezia* spp., and AEZs, whereas an insignificant (p>0.05) association was observed for the prevalence of *Eimeria* spp. and *S. papillosus*. A significant (p<0.05) association between goat age and prevalence of all GIPs was observed, where the prevalence was higher in young goats, followed by adults, and then by suckling goats. The prevalence of various GIPs was similar between male and female goats. The percentage of infection with *Eimeria* spp., *Trichuris*, *S. papillosus*, and strongyle parasitic infections was marginally higher in males than in females, whereas that of the *Moniezia* spp. was higher in females. A significant (p<0.05) association between the prevalence of *Eimeria* spp. and sampling season was observed, and there was an insignificant (p>0.05) association between the other GIPs and sampling season. The prevalence of *Eimeria* spp. infection was higher in winter (34.0%) than in summer (26.0%).

**Conclusion:**

AEZs and goat age are the most important risk factors influencing GIP infections in South African communal goats. These epidemiological parameters are important for outlining effective parasite control management systems against these GIPs in goats.

## Introduction

In the various agro-ecological zones (AEZs) of South Africa, goats are reared to fulfill multiple purposes and to meet nutritional, economic, and socio-cultural needs [1-3]. Their existence in rural households serves as a cushion in the event of crop failures due to various reasons, including climatic vagaries, especially in arid and semi-arid environments. The rich potential of the small ruminant sector, which includes goats, is not efficiently exploited due to several constraints including malnutrition, inefficient management, and diseases [4].

Goats are highly susceptible to gastrointestinal parasites (GIPs) [5,6] due to their lower innate immune response against specific helminths as a result of their evolution [7-9] and the nomadic nature of goat husbandry. In this regard, among the production constraints of goats that contribute to production losses in rural communities, GIPs constitute a major share [10,11]. The challenge is, however, much more severe in tropical countries due to favorable environmental conditions for GIP transmission [12,13], poor nutrition of the host animals [14], and poor sanitation in rural areas [15]. This makes controlling GIPs the most important health issue in goats of all ages [16-18]. The prevalence of GIP infection in livestock varies according to their existing managemental practices [19,20], season of the year [21,22], and animal age [23-25] and sex [26-28]. Several epidemiological studies have been carried out on GIPs in goat communities in South Africa [29-31], but these studies were limited in scope and coverage, sample size, and risk factors considered. Sound GIP control strategies have not yet been established with respect to the AEZs of South Africa. Therefore, it is essential to estimate the possible variation in parasitic infection in goats in different geographical regions, which could aid in designing effective control measures against parasitic diseases.

Hence, to reduce the gap in knowledge of the epidemiology of GIP infections in communal goats in different AEZs in South Africa, this study was conducted to assess the epidemiology of common gastr intestinal parasitic infections affecting goats as influenced by AEZ, sampling season, and animals’ age and sex.

## Materials and Methods

### Ethical approval

The study was approved by the Animal Research Ethics Committee of the Faculty of Science, Tshwane University of Technology [FCRE 2017/10/01 (02) (SCI)]. Ethical concerns were taken into account by adhering to the local animal welfare regulations and practices, and experiments conformed to the ethical guidelines for animal usage in the research of Tshwane University of Technology, South Africa.

### Study site and animal management

This longitudinal study was conducted in different AEZs: Arid, semi-arid, humid, and dry sub-humid zones, of KwaZulu-Natal, Limpopo, and Mpumalanga Provinces of South Africa. Goats sampled were owned by smallholder farmers who had small flock sizes ranging from one to ten animals. The selected AEZs varied in percentage of land surface, rainfall distribution, and length of the growing period. Animals were kept under extensive grazing systems where during the day they were released to graze on communal lands and corralled at night. In these areas, veterinary care was low to non-existent, and goats were not treated or dewormed. A total of 288 goats (101 male and 187 female) were randomly sampled during winter (June-July) and summer (November-December) in areas representing four AEZs (arid: 80, semi-arid: 76, humid: 62, and dry sub-humid: 70). Goats were classified by age (suckling: < 1 year, young: 1-3 years, and adult: > 3 years) as described by Kheirandish *et al*. [32]. Plastic ear tags (Allflex^®^) bearing individual identification numbers were placed on the right ear of each animal sampled to allow for repeated use of the same animals over the study period.

The sample size was determined using the following equation:


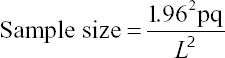


Where, n = sample size, p = expected prevalence, q = 1-p, and L = limits of error on the prevalence. Because the prevalence in the local goat population was unknown, the hypothesized prevalence of 75% was used with a 5% limit of error of the prevalence. The required sample size was calculated to be 288 goats [33].

### Fecal collection and coprology

About 10-g fecal samples from each animal were collected twice in each season directly from the rectum into airtight containers. Samples were maintained at 2-4°C in cooler boxes and were transported to the laboratory within 24 h for further coprological examination. The fecal samples were examined for the presence of GIP eggs. The fecal eggs per gram counts were determined by a modified McMaster technique, as described by Hansen and Perry [34]. The floatation fluid used was sodium chloride (NaCl: 500 g, Water: 1000 mL). The fecal samples were ground into five drops of bloat guard to prevent bubbles when counting the eggs. The GIPs were identified under a compound microscope (10×) based on the morphological appearance and size of helminth eggs, protozoa cysts, and trophozoites [35,36]. Distinguishable nematode (*Trichuris*), trematode, and cestode eggs were identified directly. A sample was considered positive when a minimum of one GIP egg was detected under the microscope. Fecal cultures were prepared by incubating 2-3 g of feces between 26°C and 28°C for 7 days at 80% humidity after which infective larvae were collected using a modified Baermann technique. L_3_-stage nematodes were identified according to the protocol proposed by Van Wyk *et al*. [37]. *Eimeria* species were identified following the sporulation of oocysts within the feces in a thin layer of 2.5% potassium dichromate for 1 week between 26°C and 28°C. The identification of *Eimeria* species was based on morphological characteristics of oocysts (size, shape, color, and presence or absence of a micropyle and its cap). The prevalence was determined using the following equation:


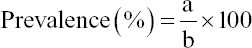


Where, “a” = Number of individuals having a disease at a particular time; “b” = Number of individuals in the population at risk at that point in time [38].

### Statistical analysis

The data were analyzed by a MiniTab17 [39] using the FREQ procedure and the association between the independent factors (AEZ, goat age and sex, and sampling season) and the prevalence of various GIP were evaluated using the Pearson Chi-square test at a significance threshold at p<0.05.

## Results

The L_3_ nematodes identified from the fecal cultures of all animals were *Haemonchus* spp., *Strongyloides papillosus*, and *Oesophagostomum* spp. in arid, semi-arid, and humid zones. *Haemonchus* and *S. papillosus* were identified in the dry sub-humid zone. Regarding *Eimeria* spp., *Eimeria arloingi*, *Eimeria christenseni*, *Eimeria alijevi*, *Eimeria jolchijevi*, *Eimeria caprina*, *Eimeria caprovina*, and *Eimeria hirci* were identified in fecal cultures from all AEZs. The prevalence of various GIPs in different AEZs of South Africa is presented in Table-1. Five types of GIP eggs observed were *Eimeria* spp., *Trichuris*, *S. papillosus*, *Moniezia* spp., and strongyle eggs, with an overall prevalence of 30.0, 26.4, 31.1, 36.6, and 59.6%, respectively. There was a significant (p<0.05) association between the prevalence of strongyles, *Trichuris*, and *Moniezia* spp. The AEZ with the highest GIP prevalence was the humid zone. There was an insignificant (p>0.05) association between the prevalence of *Eimeria* spp., *S. papillosus*, and AEZ. The nematode infections showed a preponderance of strongyle eggs, while *S. papillosus*, *Trichuris*, and *Moniezia* spp. infections were sporadic in different AEZs.

**Table-1:** Prevalence (%) of gastrointestinal parasitic infections in South African goat communities in different AEZs.

Gastrointestinal parasite	AEZ				Overall	Chi-square test	
	
	Arid (n=80)	Semi-arid (n=76)	Dry sub-humid (n=70)	Humid (n=62)		χ^2^-value	p-value
*Eimeria* spp.	27.5	26.3	33.6	33.9	30.0	3.19	0.36
*Trichuris*	22.5	21.1	28.6	35.5	26.4	8.96	0.03
*Strongyloides papillosus*	28.8	32.2	32.9	30.7	31.1	0.72	0.87
*Moniezia* spp.	27.5	42.8	40.0	37.1	36.6	8.91	0.03
Strongyles	46.9	57.2	72.1	64.5	59.6	21.5	0.00
Overall	30.6	35.9	41.4	40.3	37.1		

χ^2^=Chi-square, AEZ=Agro-ecological zones

Pearson Chi-square analysis showed a significant (p<0.05) association between the prevalence of all GIPs and goat age (Table-2). A higher prevalence of all GIPs was observed in young goats (53.8%), followed by adult goats (35.7%) and finally in suckling goats (20.0%). Strongyle infections showed a prevalence of 76.4, 61.1, and 31.8% in young, adult, and suckling goats, respectively. Sporadic infection by all GIPs was observed in goats of different ages.

**Table-2:** Prevalence (%) of gastrointestinal parasites in goats of different ages.

Gastrointestinal parasite	Goat age			Chi-square test	
	
	Suckling (n=88)	Young (n=110)	Adult (n=378)	χ^2^-value	p-value
*Eimeria* spp.	23.9	48.2	26.2	21.5	0.001
*Trichuris*	20.5	37.3	24.6	8.87	0.01
*Strongyloides papillosus*	12.5	50.0	29.9	32.8	0.001
*Moniezia* spp.	11.4	57.3	36.5	44.4	0.001
Strongyles	31.8	76.4	61.1	41.4	0.001
Overall	20.0	53.8	35.7		

χ^2^=Chi-square

There was an insignificant (p>0.05) association between the prevalence of all GIPs and the goats’ sex (Table-3). The prevalence of *Eimeria* spp., *Trichuris*, *S. papillosus*, *Moniezia* spp., and strongyle eggs in male goats was 31.7, 28.7, 34.7, 34.2, and 62.4%, respectively, while the corresponding values in females were 29.1, 25.1, 29.1, 38.0, and 58.0%, respectively.

**Table-3:** Prevalence (%) of gastrointestinal parasitic infections in goats of different sexes.

Gastrointestinal parasites	Goat sex		Chi-square test	
	
	Female (n=374)	Male (n=202)	χ^2^-value	p-value
*Eimeria* spp.	29.1	31.7	0.40	0.53
*Trichuris*	25.1	28.8	0.83	0.36
*Strongyloides papillosus*	29.1	34.7	1.86	0.17
*Moniezia* spp.	38.0	34.2	0.82	0.37
Strongyles	58.0	62.4	1.03	0.31
Means	35.9	38.3		

χ^2^=Chi-square

There was a significant (p<0.05) association between the prevalence of *Eimeria* spp. and sampling season, while there was an insignificant (p>0.05) association between the other GIPs and sampling season (Table-4). The prevalence of strongyle eggs was the highest (62.9 and 56.3% for winter and summer seasons, respectively) among the GIP infections in different seasons.

**Table-4:** Prevalence (%) of gastrointestinal parasitic infections in goats between seasons.

Gastrointestinal parasite	Season		Chi-square test	
	
	Winter (n=288)	Summer (n=288)	χ^2^-value	p-value
*Eimeria* spp.	34.0	26.0	4.37	0.04
*Trichuris*	27.4	25.4	0.30	0.59
*Strongyloides papillosus*	32.3	29.9	0.40	0.53
*Moniezia* spp.	37.5	35.8	0.19	0.67
Strongyles	62.9	56.3	2.60	0.11
Means	38.8	34.7		

χ^2^=Chi-square

## Discussion

Gastrointestinal parasitism is a major health problem affecting the productivity of goat farming worldwide [6,10,11]. The overall GIP prevalence of 37.08% observed in the present study is, however, lower than the prevalence of GIPs in goats in Cameroon (90.04%) [40], Bangladesh (77.0%) [41], and India (51.89%) [42]. Strongyle infection was the most commonly observed (64.5%) GIP infection among those observed in the present study. Our observations were similar to those of some other studies conducted in Africa and abroad, which described strongyle infection as a major problem in small ruminants [43]. However, several authors have previously reported that *Eimeria* spp. infection is the most prevalent among GIP in goats [44-46]. The present finding that the overall prevalence of *Trichuris* and *S. papillosus* was 26.4 and 31.8%, respectively, differs from the findings of Ntonifor *et al*. [40] who reported a prevalence of 13.9 and 48.9%, respectively. This difference may be attributed to different sampling sites, size and years, goat breed, and agro-climatic conditions.

The significant association observed between the AEZ and the prevalence of strongyle, *Trichuris*, and *Moniezia* spp. might be due to geographical and AEZ climatic variability, the number of animals included in various AEZs, and the management practices adopted locally. The lower prevalence of strongyles, *Trichuris*, and *Moniezia* spp. in arid zones may be attributed to the fact that these areas are extremely warm and receive scarce, erratic rainfall [47,48], which is unfavorable for GIP development, survival, and transmission [34,49].

The higher prevalence of strongyles, *Trichuris*, and *Moniezia* spp. in dry-humid and humid zones may be attributed to the fact that these areas have higher rainfall and humidity, plus moderate temperatures [47,48], which are therefore suitable for fecundity and epidemiology of the GIP. Another contributing factor may be due to poor farm management techniques and generally unhygienic farming conditions. In the present study, the prevalence of *Trichuris*, *S. papillosus*, *Moniezia*, and strongyle infections in semi-arid regions was higher than the findings in the same AEZ of Udgir, India [42]. The occurrence of *Moniezia* spp. in the tropics is associated with the ingestion of oribatid mites infected with cysticercoids of *Moniezia* spp. [40]. However, the prevalence of *Eimeria* spp. in the semi-arid zone (26.3%) was not comparable to the prevalence in semi-arid zones in India (71.45%) [46] and Ethiopia (15.7%) [50]. The prevalence of *Trichuris* spp. (21.0%) was also not comparable to that reported in the semi-arid zone in Ethiopia (0.3%) [50]. Agro-ecological factors are very important for the hatching of viable eggs, as well as parasite survival and development [51,52], leading to differences in GIP prevalence. The interactions between host factors and parasite factors determine the potential for disease/infection to occur and the pattern of infection, whereas host-environment and parasite-environment interactions influence disease transmission [53].

Infections with GIPs were more prevalent in young goats than in adult and suckling goats. Young animals are susceptible to infections due to their immunological immaturity and unresponsiveness [54], failure to separate young stock from the adult stock at the pre-weaning age, and overgrazing of infested pasture [55]. Adult animals may acquire immunity to the parasite through frequent challenges and may expel the ingested parasite before an infection is established [45,56]. The present findings agree with findings from several other studies [25,41,55], in which young goats showed a higher incidence of parasitic infection than adult goats. However, some researchers have noticed a higher prevalence in adults than in young goats [57-59].

Contrary to the present findings that goat age was significantly associated with the prevalence of GIPs, several authors [27,32,42] observed that the age of the animals was not significantly associated with the prevalence of the GIP. Furthermore, in contrast to the findings of the present study, Verma *et al*. [46] reported that suckling goats were more heavily infected with *Eimeria* and *Moniezia* spp. than young or adult goats. The overall prevalence of GIPs in suckling goats found in this study (20.0%) is, however, lower than the prevalence observed by Radfar *et al*. [60] and Dappawar *et al*. [42] who reported a prevalence of 91.42 and 45.0%, respectively, in suckling goats. The overall GIP prevalence of GIPs in adult goats in the present study was also lower than the findings of Radfar *et al*. [60] (86.8%), but was higher than the findings of Verma *et al*. [46]. The prevalence of *Trichuris* spp. in the present study was lower than that observed by Radfar *et al*. [60].

The sex-wise prevalence analysis revealed that GIP infection occurred with similar frequency in males and females, which can be attributed to the fact that both sexes are kept under similar management systems. On contrary, the significant association between goats’ sex and the prevalence of GIP was previously reported to occur more frequently in males than in females [42] or in females more than males [27,32,41]. Similar to the findings of Dappawar *et al*. [42], the percentage of *Trichuris* and *S. papillosus* infections between the two sexes was marginally higher in males as compared to females in our study. However, contrary to the findings of Dappawar *et al*. [42] and Verma *et al*. [46] who observed higher infection rates in females than in males, we observed that the prevalence of strongyles was marginally higher in males.

There was no significant association between the prevalence of *Trichuris*, *S. papillosus*, *Moniezia* spp., and strongyles during winter and summer seasons, which is noteworthy since one could anticipate that the arthropod intermediate hosts would be favored by a moist microclimate in the pasture. Similar observations were also previously reported [61]. A significant association between season and GIP prevalence was previously reported [41,42]. The significantly (p<0.05) higher prevalence of *Eimeria* spp. observed in winter could be attributed to the fact that the *Eimeria* spp. development, survival, and reproduction highly depend on seasonal factors. The nematode infections can be carried over from one favorable season to another within the host animal, which may explain the continued presence of worms in animals even during unfavorable season precluding development and survival of nematodes [62].

## Conclusion

GIPs are of economic importance and pose a great challenge to goat farmers in different AEZs. The GIPs are prevalent and endemic to a considerable extent among the goats in the study area of South Africa. AEZ and goat age were the most important factors influencing the risk of GIP infections in South African goat communities. These risk factors need to be taken into consideration when designing effective parasite control management systems for these animals. The present study validates the need to create awareness to develop the appropriate educational materials for goat farmers and the general public regarding infection potential, the dangers these GIPs pose, and possible ways to combat them. Further detailed investigations into the infestation intensity and patterns, as well as other epidemiological factors, should be carried out to generate a comprehensive parasite profile of South African goat community.

## Authors’ Contributions

This study is the component of the work toward the Ph.D. thesis of the first author TJM, under the guidance of KAN and BM. TJM designed the study, collected, and analyzed the data, and wrote the manuscript. KAN and BM designed the study, coordinated the work, and revised the manuscript. All authors read and approved the final manuscript.

## References

[1] Homann S, Van Rooyen A, Moyo T, Nengomasha Z (2007). Goat production and marketing:Baseline information for semi-arid Zimbabwe. ICRISAT.

[2] Ruto E, Garrod G, Scarpa R (2008). Valuing animal genetic resources:A choice modeling application to indigenous cattle in Kenya. J. Agric. Econ.

[3] Sweet R.J (2008). Managing and developing African Pastoralism:Some practical considerations. Grassroots.

[4] Teklye B, Epidemiology of endoparasites of small ruminants in Sub-Saharan Africa (1991).

[5] Mandonnet N, Aumont G, Fleury J, Arquet R, Varo H, Gruner L, Bouix J, Khang J.V (2001). Assessment of genetic variability of resistance to gastrointestinal nematode parasites in Creole goats in the humid tropics. J. Anim. Sci.

[6] Sissay M, Asefa A, Uggla A, Waller P (2006). Anthelmintic resistance of nematode parasites of small ruminants in eastern Ethiopia:Exploitation of refugia to restore anthelmintic efficacy. Vet. Parasitol.

[7] Pomroy W.E, Lambert M.G, Betteridge K (1986). Comparison of fecal strongylate egg counts of goats and sheep on the same pasture. N. Z. Vet. J.

[8] Mckenna P.B, Watson T.G (1987). The comparative efficacy of four broad-spectrum anthelmintics against some experimentally induced trichostrongylid infections in sheep and goats. N. Z. Vet. J.

[9] Hoste H, Torres-Acosta J.F, Aguilar-Caballero A.J (2008). Nutrition parasite interactions in goats:Is immunoregulation involved in the control of gastrointestinal nematodes?. Parasite Immunol.

[10] Sutherland I, Scott I (2010). Gastrointestinal Nematodes of Sheep and Cattle: Biology and Control.

[11] McRae K.M, Mcewan J.C, Dodds K.G, Gemmell N.J (2014). Signatures of selection in sheep bred for resistance or susceptibility to gastrointestinal nematodes. BMC. Med. Genomics.

[12] Mohanta U.K, Anisuzzaman T, Das P.M, Majumder S, Mondal M.M.H (2007). Prevalence, population dynamics and pathological effects of intestinal helminths in black Bengal goats. Bangladesh J. Vet. Med.

[13] Zeryehun T (2012). Helminthosis of sheep and goats in and around Haramaya, southeastern Ethiopia. J. Vet. Med. Anim. Health.

[14] Mbuh J.V, Ndamukong K.J.N, Ntonofor N, Hforlem G.F (2008). Parasites of sheep and goat and their prevalence in Bokova, a rural area of Buea subdivision, Cameroon. Vet. Parasitol.

[15] Badran I, Abuamsha R, Aref R, Alqisi W, Alumor J (2012). Prevalence and diversity of gastrointestinal parasites in small ruminants under two different rearing systems in Jenin district of Palestine. An. Najah Uni. J. Res.

[16] Nye T.L, Moore R (2004). Meat Goat Production and Budgeting Ohio line.

[17] Hoste H, Jackson F, Athanasiadou S, Thamsborg S.M, Hoskin S.O (2006). The effects of tannin-rich plants on parasitic nematodes in ruminants. Trends Parasitol.

[18] Waller P.J (2006). Sustainable nematode parasite control strategies for ruminant livestock by grazing management and biological control. Anim. Feed Sci. Tech.

[19] Pal R.A, Qayyum M (1992). Breed, age and sex-wise distribution of gastro-intestinal helminths of sheep and goats in and around Rawalpindi region. Pak. Vet. J.

[20] Kumar B, Maharana B.R, Prasad A, Joseph P.J, Patel B, Patel J.S (2016). Seasonal incidence of parasitic diseases in bovines of South Western Gujarat, India. J. Parasit. Dis.

[21] Godara R, Katoch R, Yadav A, Rastogi A (2014). Epidemiology of paramphistomosis in sheep and goats in Jammu, India. J. Parasit. Dis.

[22] Khajuria J.K, Katoch R, Yadav A, Godara R, Gupta S.K, Singh A (2012). Seasonal prevalence of gastrointestinal helminths in sheep and goats of middle agro-climatic zone of Jammu province. J. Parasit. Dis.

[23] Lone B.A, Chishti M, Ahmad F, Tak H (2012). A survey of gastrointestinal helminth parasites of slaughtered sheep and goats in Ganderbal, Kashmir. Glob. Vet.

[24] Ayaz M.M, Raza M.A, Murtaza S, Akhtar S (2013). Epidemiological survey of helminths of goats in Southern Punjab, Pakistan. Trop. Biomed.

[25] Zvinorova P.I, Halimani T.E, Muchadeyi F.C, Matika O, Riggio V, Dzama K (2016). Prevalence and risk factors of gastrointestinal parasitic infections in goats in low-input low-output farming systems in Zimbabwe. Small Rumin. Res.

[26] Raza M.A, Iqbal Z, Jabbar A, Yaseen M (2007). Point prevalence of gastrointestinal helminthiasis in ruminants in southern Punjab, Pakistan. J. Helminthol.

[27] Emiru B, Ahmed Y, Tigre W, Feyera T, Deressa B (2013). Epidemiology of gastrointestinal parasites of small ruminants in Gechi district, Southwest Ethiopia. Adv. Biomed. Res.

[28] Vieira V.D, Feitosa T.F, Vilela V.L.R, Azevedo S.S, de Almeida Neto João Leite de Morais D.F, Ribeiro A.R.C, Athayde A.C.R (2014). Prevalence and risk factors associated with goat gastrointestinal helminthiasis in the Sertão region of Paraíba state, Brazil. Trop. Anim. Health Prod.

[29] Boomker J, Horak I.G, Ramsay K.A (1994). Helminth and arthropod parasite of indigenous goats in the Northern Transvaal. Onderstepoort J. Vet. Res.

[30] Tsotetsi A.M, Mbati P.A (2003). Parasitic helminths of veterinary importance in cattle, sheep and goats on communal farms in the northeastern free state, South Africa. J. S. Afri. Vet. Assoc.

[31] Gwaze F.R, Chimonyo M, Dzama K (2009). Communal goat production in Southern Africa:Review. Trop. Anim. Health Prod.

[32] Kheirandish R, Nourollahi-Fard S.R, Yadegari Z (2014). Prevalence and pathology of coccidiosis in goats in Southeastern *Iran*. J Parasit. Dis.

[33] Thrusfield M (1997). Veterinary Epidemiology.

[34] Hansen J, Perry B (1994). Epidemiology, Diagnosis and Control of Helminth Parasites of Ruminants.

[35] Foriet W (1999). Reference Manual of Veterinary Parasitology.

[36] Zajac M, Conboy G (2006). Veterinary Clinical Parasitology.

[37] Van Wyk J.A, Cabaret J, Michael L.M (2004). Morphological identification of nematodes of small ruminants and cattle simplified. Vet. Parasitol.

[38] Thrusfield M (2005). Veterinary Epidemiology.

[39] MiniTab 17 Statistical Software (2017). Computer Software.

[40] Ntonifor H, Shei S, Ndaleh N, Mbunkur G (2013). Epidemiological studies of gastrointestinal parasitic infections in ruminants in Jakiri, Bui division, North West region of Cameroon. J. Vet. Med. Anim. Health.

[41] Islam M.S, Hossain M.S, Dey A.R, Alim M.A, Akter S, Alam M.Z (2017). Epidemiology of gastrointestinal parasites of small ruminants in Mymensingh, Bangladesh. J. Adv. Vet. Anim. Res.

[42] Dappawar M.K, Khillare B.S, Narladkar B.W, Bhangal G.N (2018). Prevalence of gastrointestinal parasites in small ruminants in Udgir area of Marathwada. J. Entomol. Zool. Stud.

[43] Fikru R, Teshale S, Reta D, Yosef K (2006). Epidemiology of gastrointestinal parasites of ruminant in Western Oromia, Ethiopia. Inter. J. Appl. Res. Vet. Med.

[44] Jatau I.D, Abdulganiyu A, Lawal A.I, Okubanjo O.O, Yusuf K.H (2011). Gastrointestinal and hemoparasitism of sheep and goats at slaughter in Kano, Northern-Nigeria, Sokoto. J. Vet. Sci.

[45] Singh A.K, Das G, Roy B, Nath S, Naresh R, Kumar S (2015). Prevalence of gastrointestinal parasitic infections in goat of Madhya Pradesh, India. J. Parasit. Dis.

[46] Verma R, Sharma D, Paul S, Kumaresan G, Dige M, Kumar S.V, Kumar R.P, Bhusan S, Banerjee S.P (2018). Epidemiology of common gastrointestinal parasitic infections in goats reared in semi-arid region of India. J. Anim. Res.

[47] Schulze R.E (1997). South African Atlas of Agrohydrology and Climatology. Water Research Commission, Pretoria Report TT82/96.

[48] Hunters R.A, Buck N, Jarrige R, Beranger C (1992). Nutrition and climatic limits of beef production in the tropics. World Animal Science. C5. Beef Cattle Production.

[49] Kumsa B, Abebe G (2009). Multiple anthelmintic resistance on a goat farm in Hawassa (Southern Ethiopia). Trop. Anim. Health Prod.

[50] Dabasa G, Shanko T, Zewdei W, Jilo K, Gurmesa G, Abdela N (2017). Prevalence of small ruminant gastrointestinal parasites infections and associated risk factors in selected districts of Bale zone, southeastern Ethiopia. J. Parasitol. Vector Biol.

[51] Odoi A, Gathuma J.M, Gachuiri C.K, Omore A (2007). Risk factors of gastrointestinal nematode parasite infections in small ruminants kept in smallholder mixed farms in Kenya. BMC Vet. Res.

[52] Ratanapob N, Arunvipas P, Kasemsuwan S, Phimpraphai W, Panneum S (2012). Prevalence and risk factors for intestinal parasite infection in goats raised in Nakhon Pathom province, Thailand. Trop. Anim. Health Prod.

[53] Roeber F, Jex A.R, Gasser R.B (2013). Impact of gastrointestinal parasitic nematodes of sheep, and the role of advanced molecular tools for exploring epidemiology and drug resistance-an Australian perspective. Parasit. Vectors.

[54] Asanji M.F, Williams M (1987). Variables affecting the population dynamics of gastrointestinal helminth parasites of small ruminants in Sierra leone. Bull. Anim. Health Prod.

[55] Ndamukong K.N.J (1985). *Strongyle* infestations of sheep and goats at Mankon station, Mankon station, Bamenda, Cameroon. Vet. Parasitol.

[56] Shah-Fischer M, Say R (1989). Manual of Tropical Veterinary Parasitology. CAB International; the Technical Center for Agricultural and Rural Cooperation (CTA).

[57] Uddin M.Z, Farjana T, Begum N, Mondal M.M.H (2006). Prevalence of amphistomes in Black Bengal goats in Mymensingh district. Bangladesh J. Vet. Med.

[58] Hassan M.M, Hoque M.A, Islam S.K.M, Khan S.A, Roy K, Banu Q (2011). A prevalence of parasites in black Bengal goats in Chittagong, Bangladesh. Int. J. Livest. Prod.

[59] Admasu P, Nurlign L (2014). Prevalence of gastrointestinal parasites of small ruminants in Kuarit district, North West Ethiopia. Afr. J. Bas. Appl. Sci.

[60] Radfar M.H, Sakhaee E, Bafti M.S, Mohammadi H.H (2011). Study on gastrointestinal parasitic infections of Raeini goats. Iran. J. Vet. Res.

[61] Sissay M.M, Uggla A, Waller P.J (2007). Prevalence and seasonal incidence of nematode parasites and fluke infections of sheep and goats in Eastern Ethiopia. Trop. Anim. Health Prod.

[62] Nwosu C.O, Madu P.P, Richards W.S (2007). Prevalence and seasonal changes in the population of gastrointestinal nematodes of small ruminants in the semi-arid zone of North-Eastern Nigeria. Vet. Parasitol.

